# Vitamin B12 in Pregnancy and its Relationship with Maternal BMI and Gestational Diabetes Mellitus - A study from Pakistan

**DOI:** 10.12669/pjms.38.4.5120

**Published:** 2022

**Authors:** Aasia Kanwal, Aisha Bashir

**Affiliations:** 1 Dr. Aasia Kanwal, M.Phil. Physiology, Department of Physiology, Shalamar Medical & Dental College, Lahore, Pakistan; 2 Dr. Aisha Bashir, M.Phil. Physiology, Department of Physiology, Shalamar Medical & Dental College, Lahore, Pakistan

**Keywords:** Gestational diabetes mellitus, BMI, Vitamin B12, Maternal obesity

## Abstract

**Objectives:**

To determine the levels of vitamin B12 in pregnant women and to explore the relationship of vitamin B12 with maternal BMI and gestational diabetes mellitus.

**Methods:**

This cross-sectional study was conducted at Department of Physiology, UHS, Lahore from February 2019 to March 2020. Ninety pregnant women in early third trimester of pregnancy were selected. Forty-five women had gestational diabetes and forty-five were healthy pregnant women. Serum vitamin B12 and fasting blood glucose (FBG) levels were determined by ELISA and glucose oxidase method respectively. 1^st^ trimester BMI was also recorded. SPSS version 23 was used to analyze the data.

**Results:**

Serum vitamin B12 was significantly lower in GDM group (149.41±13.66) as compared to non-GDM group (357.49±42.07). BMI was significantly higher in GDM group (32.94±2.10) as compared to non-GDM group (23.52±1.83). Significant negative correlation (-0.78**) was observed between 1^st^ trimester BMI and vitamin B12 in late pregnancy. Regression analysis revealed that high BMI was associated with decrease in vitamin B12 and increased risk of gestational diabetes mellitus (3.26***). Moreover, vitamin B12 was partially mediating the relationship between BMI and FBG.

**Conclusion:**

Low vitamin B12 levels have an association with maternal BMI and gestational diabetes mellitus.

## INTRODUCTION

Obesity is rapidly rising among pregnant and women of childbearing age. In United Kingdom 20% of the pregnancies are affected by obesity.^1^ Overweight and obesity is reported in 38.4% women of childbearing age in Pakistan. While in United States it is reported to be 61.9%.^2^ Overweight and obesity increase the risk of gestational diabetes mellitus.^3^,^4^ Dietary deficiencies are common among pregnant women and may lead to adverse fetal and maternal outcomes in pregnant women with high BMI.^5^

Vitamin B12 (Vit B12) an antioxidant vitamin, is required for Remethylation of homocysteine and to convert methyl malonyl CoA into succinyl CoA.^6^ There is increased risk of Vit B12 deficiency in pregnant women due to insufficient consumption and increased metabolic demands. Deficiency is frequent in all trimesters of pregnancy.^7^ Low Vit B12 levels cause elevated homocysteine and methyl malonyl CoA levels and the later can impair fat catabolism and promote lipid production.^7^,^8^ So, Vit B12 deficiency may cause obesity and decreased sensitivity to insulin in pregnant women.^9^ Low Vitamin B12 levels have negative relationship with BMI.^10^ Vitamin B12 deficiency can also occur secondary to overcooking of meat.^11^

The key objective of our study was to determine vitamin B12 levels in pregnancy and to explore the association of vitamin B12 with maternal BMI and gestational diabetes mellitus.

## METHODS

A cross sectional comparative study of the pregnant women attending the outpatient department of different government sector hospitals of Lahore was conducted from 2019 to 2020. Institutional review board of University of Health Sciences, Lahore granted the approval of study (No. UHS/REG-19/ERC/401). Sample size was calculated to be 60 (30 in each group) by using 5% level of significance and 90% power of study, but 90 pregnant women were sampled to increase the statistical power for the study.

The study population includes 90 pregnant women among which 45 had gestational diabetes and 45 were without gestational diabetes mellitus. The diagnostic criteria used for gestational diabetes mellitus was 75g OGTT, defined by American Diabetes Association (ADA). Pregnant women with previous history of gestational diabetes, grand multiparity, liver and kidney disease, and taking vitamin B12 and folate supplementation within two weeks of sampling were excluded from the study. Detailed history of the pregnant women including dietary and supplementation history along with 1^st^ trimester BMI was recorded.

The study population was sampled in early third trimester of pregnancy after getting written consent. Three milliliters of venous blood were drawn from the antecubital veins of the subjects after an overnight fast of 8 to 12 hours following aseptic technique. Blood was centrifuged to extract the serum for glucose and vitamin B12 analysis. Serum glucose levels were determined by glucose oxidase method. Serum was stored at -80ºC within four hours of collection and thawed just before analysis. Serum Vitamin B12 levels were determined by ELISA (Bioassay Technology Laboratory, Shanghai, China). Microsoft Excel sheets were used for data entry. The data was analyzed by using SPSS version 23.

Group comparison between GDM and non-GDM group was done by independent sample T test as data was normally distributed. Pearson correlation test was used to establish the correlation between variables. Median regression analysis was done to check causal association of the variables.

## RESULTS

The two groups were matched for age (p=0.21) and gestational age (p=0.18). There was statistically significant difference in fasting blood sugar levels in two groups (p=0.000). Vit B12 levels were significantly lower in GDM group (149.41±13.66) as compared to non-GDM group (357.49±42.07) at (p=0.000). Serum homocysteine levels were significantly higher in GDM group (16.95±5.70) compared to non-GDM group (10.05±3.35) at (p=0.030). Mean BMIs were significantly higher in in GDM group (32.94±2.10) as compared to non-GDM group (23.52±1.83) at (p=0.000) as presented in Table-I.

**Table-I:** Group comparison of GDM and Non-GDM Group.

	GDM (n=45)	Non-GDM (n=45)	

Variables	Mean ± SD	Mean ± SD	Significance
Age (Years)	31.02 ± 3.83	30.10 ± 3.49	0.210
BMI (Kg/m^2^)	32.94 ± 2.10	23.52 ± 1.83	0.000
G. Age (Weeks)	30.05 ± 1.48	30.29 ± 1.38	0.181
FBG (mg/dL)	138.96 ± 12.66	84.07 ± 6.22	0.000
VB (pg/mL)	149.41 ± 13.66	357.49 ± 42.07	0.000
Homocysteine (μmol /L)	16.95 ± 5.70	10.05 ± 3.35	0.030
Family history of T2DM	20 (66)	3 (10)	0.744

SD: Standard Deviation, BMI: Body Mass Index, G. Age: Gestational Age, VB: Vitamin B12, FBG: Fasting Blood Glucose, T2DM: Type 2 diabetes mellitus.

*p-value < 0.05 is considered statistically significant.

BMI had a significant positive correlation with fasting blood glucose (0.32***) and significant negative correlation with vitamin B12 (-0.78**). Vitamin B12 had significant negative correlation with fasting blood glucose (-0.28**) as shown in Table-II.

**Table-II:** Correlation Analysis GDM Group.

Variables	BMI	VB	FBG
BMI		-0.78**	0.326***
VB	-0.78**		-0.28**
FBG	0.326***	-0.28**	

***Notes:*** Vitamin B12 and fasting blood glucose are denoted by VB and FBG, respectively. Whereas, correlation coefficients, significant at <0.01 level and <0.05 level was presented with *** and **, respectively.

The results for mediation analysis is shown in Table-III. In the second column, estimates were presented for regressing FBG on BMI is notable that BMI has a highly significant and positive impact on FBG, whereas a single unit increase in BMI can result into a 4.96 increase in BSF. While the impact of BMI on Vitamin B12 is negative, as given in the third column of Table-III. Forth column states a negative beta if blood sugar fasting is regressed upon Vitamin B12, the mediator. The significant results of aforementioned three relationships validates the prerequisites of mediation regression as per Barron and Kenny 1986. The impact of independent variable BMI highly significant yet the BMI and Vitamin B12 are simultaneously taken at independent side of regression. These results reveal that Vitamin B12 has a partial mediation role between BMI and FBG. Finally, the analysis was controlled by including the baseline variables. As presented in the extreme right column, it can be noted that the results were robust against the inclusion of control variables.

**Table-III:** Mediation Analysis in GDM group.

	BMI → FBG	BMI → VB	VB → FBG	BMI + VB → FBG	BMI + VB + BS → FBG
BMI	4.96*** (0.549)			4.71*** (0.617)	3.26*** (0.70)
VB		-2.93** (.929)	-0.411** (0.133)	0.384** (0.137)	0.019** (0.11)
Age					-0.368 (0.31)
G. Age					1.09** (0.32)
Family History of DM					-0.27** (0.12)
Constant	-24.415* (18.132)	230.03*** (30.67)	193.82*** (17.85)	-5.04* (28.41)	55.04** (23.05)
R^2^	0.421	0.204	0.197	0.683	0.615
Adjusted R^2^	0.367	0.184	0.176	0.666	0.635

FBG: Fasting blood glucose, VB: Vitamin B12, G. Age: Gestational age, DM: Diabetes Mellitus

Notes: Figures in the parenthesis indicate the standard error of beta coefficient. While the beta coefficient significant at 1%, 5% and 10% levels were represented by ***, **, * respectively.

## DISCUSSION

The main findings of this study have demonstrated that high BMI in early first trimester of pregnancy is associated with low B12 levels in late pregnancy and low vitamin B12 is a risk factor for GDM. There is extensive data available linking vitamin B12 with insulin resistance, but the limited and controversial data exists finding the association of vitamin B12 with obesity and GDM. Butt et al., 2017 explored the relationship of B12 with GDM among Pakistani pregnant women, though they excluded BMI while establishing this relationship.^12^ Correspondingly, Krishnaveni et al., also reported significant association of low B12 levels with GDM, but these patients had lower BMIs.^13^ Our study observed significantly lower levels of B12 in GDM group just like an Indian and British study.^13^,^14^ They explored this link in a population with different lifestyle, dietary behavior, ethnicity, and nutritional standards. It can be inferred that B12 deficiency is also common in other populations.

The study by Sukumar et al., conducted on 344 British pregnant women using the 75g OGTT criteria defined by ADA reported the significant association of obesity with B12 deficiency and risk of GDM at 28 to 32 weeks of gestation just like our study.^14^ A local study conducted in Muzaffarabad reported that the rate of gestational diabetes was higher (27.3% vs. 9.1%) in obese pregnancies as compared to non-obese pregnancies (p-value< 0.05) though this study did not determine vitamin B12 levels in these women.^15^ Maternal BMI at the early stage of pregnancy may affect maternal and fetal outcomes later on.

A study by Li, S et al., involving 406 Chinese pregnant women using the International Association of Diabetes and Pregnancy Study Groups (IADPSG) criteria, observed high risk of GDM in pregnancies with low Vit B12 and high BMIs.^16^

### Strength &Limitations of the study:

This is the first study from Pakistan to report that Vit B12 deficiency is common in pregnant women with obesity and linked with gestational diabetes mellitus. Limitations include the sample size, secondly it was cross sectional study that cannot establish causal association. GDM and obesity are growing problems, we recommend longitudinal studies with larger sample size to explore this relationship of vitamin B12 with obesity and GDM, that may help manage GDM with simple lifestyle modifications and B12 supplementation.

## CONCLUSION

Low vitamin B12 levels have an association with maternal BMI and gestational diabetes mellitus.

### Authors Contribution:

**AK:** Conceived, designed, collected data, and did statistical analysis & editing of manuscript, is responsible for integrity of research.

**AB:** Did data collection and manuscript writing.
